# New records in non-native vascular plants of Russian Lapland

**DOI:** 10.3897/BDJ.10.e78166

**Published:** 2022-01-06

**Authors:** Mikhail Kozhin, Alexander Sennikov

**Affiliations:** 1 Avrorin Polar-Alpine Botanical Garden-Institute, Apatity, Russia Avrorin Polar-Alpine Botanical Garden-Institute Apatity Russia; 2 Kandalaksha Strict Nature Reserve, Kandalaksha, Russia Kandalaksha Strict Nature Reserve Kandalaksha Russia; 3 University of Helsinki, Helsinki, Finland University of Helsinki Helsinki Finland; 4 Komarov Botanical Institute, Saint-Petersburg, Russia Komarov Botanical Institute Saint-Petersburg Russia

**Keywords:** alien species, human introduction, invasion status, Murmansk Region, naturalisation, Russia

## Abstract

**Background:**

The non-native vascular plants of Murmansk Region (European Russia) are under active investigation towards the compilation of the first complete checklist. This work is part of the project 'Flora of Russian Lapland', which ultimately aims at the complete inventory of the taxonomy, distribution and status of vascular plant species in Murmansk Region, based on the comprehensive database of herbarium specimens, field observations and literature.

**New information:**

New territory-level records of non-native vascular plants emerged during our inventory of herbarium collections and recent fieldwork. Fourteen species (*Anthemisruthenica*, *Aruncusdioicus*, *Bromuscommutatus*, *Chaerophyllumhirsutum*, *Galegaorientalis*, *Geumaleppicum*, *Leonurusquinquelobatus*, *Lepidiumdensiflorum*, *Levisticumofficinale*, *Myrrhisodorata*, *Phleumphleoides*, *Prunusarmeniaca*, *Rorippasylvestris*, *Seneciovernalis*) are reported as new to Murmansk Region. The historical occurrences of alien plants appeared in the territory largely as contaminants (of seed or forage). In particular, *Rorippasylvestris* and *Seneciovernalis* arrived with the forage imported during the Second World War. All recent occurrences originated by escape from confinement (ornamental purposes, horticulture, agriculture), reflecting a high diversity of the modern assortment of cultivated plants in commerce and private gardens. Regarding the invasion status, five alien species are considered casual and eight species are treated as locally established or persisting (for uncertain time). Only one species, *Galegaorientalis*, is considered naturalised and capable of further spreading in the territory, although without invasive potential.

## Introduction

Non-native plants have become a serious issue at the global scale, disturbing native ecosystems, human well-being and economy (Simberloff et al. 2012). With the rising awareness about the negative impact caused by unwanted plant introductions, many European countries performed inventories of alien plants and their invasive status. Among such achievements of the latest decade, the checklists for Greece (Arianoutsou et al. 2010), Portugal (Doningues de Almeida and Freitas 2006, Domingues de Almeida and Freitas 2012), Albania (Barina et al. 2014), Turkey (Uludag et al. 2017), Italy (Galasso et al. 2018) and Belgium (Verloove et al. 2020) have been compiled or significantly updated. This progress has allowed for a new analysis of introduction pathways, gateways and time trends of alien plants in Europe (Arianoutsou et al. 2021).

The number and percentage of non-native vascular plants in Europe is overwhelming. According to the latest continent-scale inventory (Pyšek et al. 2009), there were 5,789 alien plant species in Europe, of which 2,843 were alien to Europe. The statistics published for individual territories or countries in Northern Europe (including northern European Russia) are also impressive: 558 alien plant species were recorded in the Russian European Arctic lowland (Wasowicz et al. 2019), 788 (44%) alien plant species were known in Russian Karelia (Kravchenko 2007), and 1828 (65%) alien plant species were counted in Finland (Kurtto et al. 2019).

**Figure 1.**
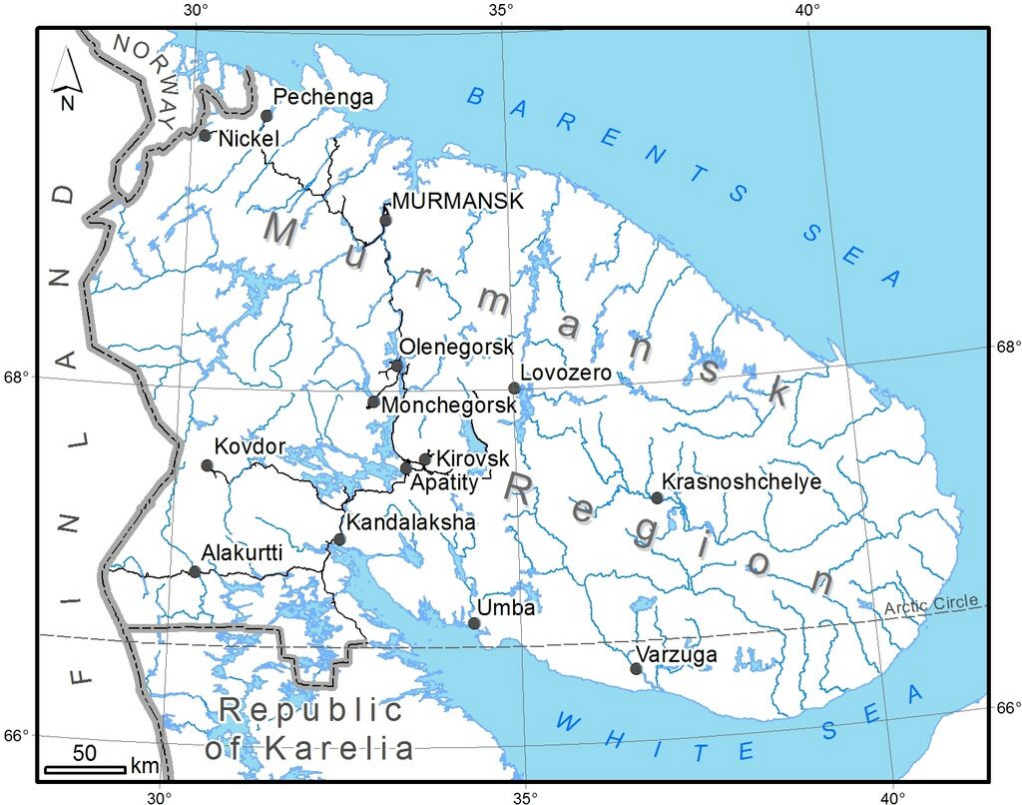
Study area: Murmansk Region, Russia.

Murmansk Region is an administrative territory (top-level federal subject) situated in the north-western part of European Russia (Fig. 1). Its area constitutes 144,902 km^2^, thus exceeding in size some European countries like Greece. Geographically, Murmansk Region occupies the Kola Peninsula with the neighbouring mainland, being surrounded by the Barents Sea in the north and the White Sea in the south and east. It is situated within the Subarctic Zone, almost completely north of the Arctic Circle, and is covered by the transition gradient from tundra along the northern coast, through forest tundra in the major part of the mainland, to northern taiga in the south (Chernov 1971). Two significant mountain ranges are present in the territory, Chibiny and Lovozero, with the maximum height reaching 1191 m.

The vascular plants of Murmansk Region have been in focus for over 150 years; this territory received its first (albeit very provisional) checklist already in 1831 (Fellman 1831), which was professionally updated in less than 40 years (Fellman 1869) and the whole flora was treated in a full-size, detailed, multivolume academic publication during 1953-1966 (Gorodkov 1953, Poyarkova 1954, Poyarkova 1956, Poyarkova 1959, Poyarkova 1966). All species accounts in this book were accompanied by point distribution maps, which have been recently digitised and made available through GBIF (Kozhin et al. 2020a). This information was updated in Ramenskaya and Andreeva (1982) and Ramenskaya (1983). Rare and endangered native plants were studied and mapped for the Red Data Book of Murmansk Region, which has been recently updated (Konstantinova et al. 2014).

Despite this impressive progress in floristic studies, non-native vascular plants of Murmansk Region have never been completely inventoried. The last published synopsis of vascular plants of this territory (Ramenskaya 1983) included only 270 alien species, whereas the latest provisional count (Kozhin et al. 2020) suggested that the number of alien plant species in this territory may have reached 502 (less than 50% of the total flora). This means that the information on nearly 50% of non-native vascular plants registered in the territory is hidden in a number of minor paper publications that belong to the vast corpus of Russian grey botanical literature, which has never been indexed and remains poorly accessible even to experts in the field.

In course of preparation of the first checklist of non-native vascular parts in Murmansk Region (part of the project 'Flora of Russian Lapland'), we continued our fieldwork and survey of herbarium collections in order to enhance the database of occurrences of the vascular plants. While cataloguing the collections at the University of Helsinki (H), barcoding the collections at the Avrorin Polar-Alpine Botanical Garden-Institute (KPABG) and scanning the collections at the Komarov Botanical Institute (LE), we discovered a few specimens which had previously escaped the attention of botanists working with the flora of the region.

Among the unfiled collections kept at Avrorin Polar-Alpine Botanical Garden-Institute (KPABG), we have found a large set of herbarium specimens which provided the documentation to the inventory of weedy plants in Russian Lapland made by E.V. Shlyakova in the 1950s and the early 1960s. She extensively studied weeds in Murmansk Region (Shlyakova 1958, Shlyakova 1961) and other parts of the boreal zone of the European part of the USSR; this work culminated in a manual that summarised this knowledge (Shlyakova 1982). Although the collection was revised by the author, we have found some specimens misidentified or left unsorted without identifications, which contained a few weedy plants previously unrecognised in Murmansk Region. These records are also reported here.

The present contribution is a complement to Kozhin et al. (2020), with a greater focus on the historical information derived from herbarium collections and a smaller proportion of new records from the recent fieldwork. Since the checklist of non-native plants of Murmansk Region has not been compiled and the background data have not been analysed yet, we are not able to present a detailed analysis of the new data in a broader context. We believe that this analysis will follow in the nearest future.

## Materials and methods

**Figure 2.**
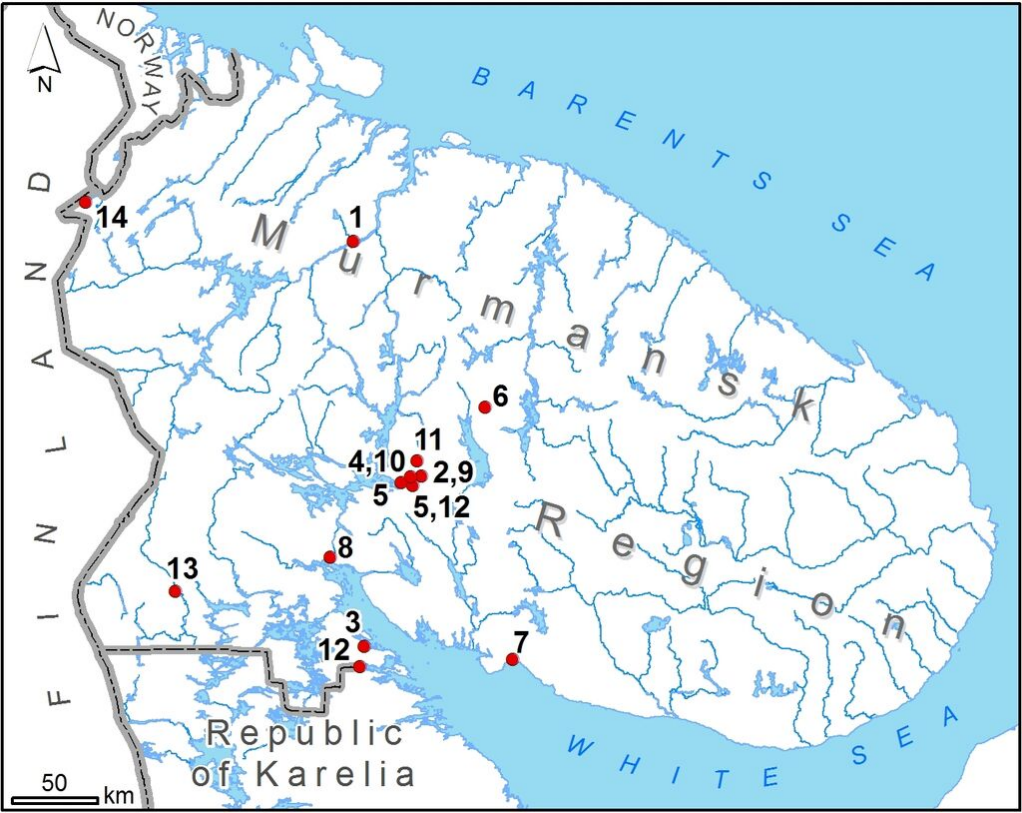
Collection localities of newly recorded alien plants in Murmansk Region, Russia. 1 — *Anthemisruthenica*, 2 — *Aruncusdioicus*, 3 — *Bromuscommutatus*, 4 — *Chaerophyllumhirsutum*, 5 — *Galegaorientalis*, 6 — *Geumaleppicum*, 7 — *Leonurusquinquelobatus*, 8 — *Lepidiumdensiflorum*, 9 — *Levisticumofficinale*, 10 — *Myrrhisodorata*, 11 — *Phleumphleoides*, 12 — *Prunusarmeniaca*, 13 — *Rorippasylvestris*, 14 — *Seneciovernalis*.

The new information on non-native vascular plants in Murmansk Region was collected by M.N. Kozhin in the field during his work on the project 'Flora of Russian Lapland' in 2018-2020 (Fig. 2). The towns of Apatity and Kirovsk, as well as some villages along the southern and northern coasts of the Kola Peninsula were surveyed for alien plants.

In compilation and verification of the first checklist of non-native vascular plants of Murmansk Region (in prep.), all available herbarium collections at H, KPABG and LE (acronyms according to Thiers 2021) were screened for occurrences in this territory. Some previously unpublished records were identified or verified by the authors and included in this list.

Species are treated according to the methodology and data structure similar to that employed by Sennikov and Lazkov (2021). The status of non-native plant species was determined following the definitions proposed by Richardson et al. (2000) and Pyšek et al. (2004). The historical information was examined to uncover the history and pathways of introduction of a certain plant species to a certain locality. The pathways of introduction were coded according to Hulme et al. (2008) and Harrower et al. (2018). The historical periodisation followed major events of the political history of the territory. The distributional information was largely derived from PoWO (2021) and various taxonomic authorities. Major reference sources were used to assess the distribution and status of non-native taxa in the neighbouring territories (Hämet-Ahti et al. 1998, Tzvelev 2000, Kravchenko 2007). Life forms are briefly characterised according to the Raunkiær system (Raunkiaer 1905, Raunkiaer 1937) and the Serebryakov system (Serebryakov 1962, Zhmylev et al. 2017).

## Data resources

The specimen information was deposited in the Flora of Russian Lapland Database (https://laplandflora.ru/). The herbarium specimens were deposited and partly imaged at H, INEP, KAND, KPABG, MW (https://plant.depo.msu.ru/) and LE (http://en.herbariumle.ru/). The new records were georeferenced and made available through GBIF within curatorial datasets (Kozhin and Sennikov 2020, Kozhin 2021, Kozhin and Sennikov 2021, Lampinen and Laiho 2021, Seregin 2021). This information is also available for download here (Suppl. material 1).

## Taxon treatments

### Anthemis ruthenica

M.Bieb.

409FCBEE-7947-5624-89B7-C71886B2B992

urn:lsid:ipni.org:names:177582-1

https://laplandflora.ru/#28334

https://www.gbif.org/occurrence/3400181303

Anthemis ruthenica M.Bieb., Fl. Taur.-Caucas. 2: 330 (1808).

#### Distribution

##### Native distribution

Europe (temperate), Mediterranean, Caucasus.

##### Secondary distribution

Europe (north), Asia.

##### Distribution in neighbouring territories

In Karelia, the species is known as casual on railways (Kravchenko 2007). In Finland, it is known as casual alien in many places in the south and seldom in the north, mostly introduced with transport (Kurtto 1998b).

##### New record

Russia. Murmansk Region. Kola District. Tuloma Village, state farm 'Tuloma', field no. 1, cultivated field of perennial grasses, 31.08.1953, *E. Shlyakova* #36 (KPABG 042732).

##### Pathways of introduction

Transport – Contaminant: Seed contaminant.

The species was found on fields, thus indicating its arrival with contaminated seed.

##### Period of introduction

USSR, after the Second World War (1945-1991).

This casual alien has not been known prior to the period of its first record and had hardly had a chance for longer survival in the agricultural habitats.

##### Invasion status

Historical casual occurrence. No new records, apparently extinct in the territory.

#### Ecology

Sands, rock outcrops, disturbed ground.

#### Biology

Annual. Therophyte with taproot.

### Aruncus dioicus

(Walter) Fernald

AF85FB1F-E30D-5C5B-971B-9F41DF11ACC5

urn:lsid:ipni.org:names:20973-2

https://laplandflora.ru/#19949

https://www.gbif.org/occurrence/3400181306

Aruncus dioicus (Walter) Fernald, Rhodora 41: 423 (1939) - *Actaeadioica* Walter, Fl. Carol.: 152 (1788).
Aruncus dioicus *Aruncussylvester*
Aruncus dioicus *Aruncusasiaticus*

#### Distribution

##### Native distribution

Europe (temperate), Caucasus, Northern Asia (south Siberia, east Mongolia), Himalayas, China, South-Eastern Asia.

##### Secondary distribution

Commonly cultivated for ornamental purposes and occasionally runs wild in Europe and North America.

##### Distribution in neighbouring territories

Seldom runs wild in North-Western European Russia (Tzvelev 2000).

##### New record

Russia. Murmansk Region. Kirovsk District. Highway Apatity - Kirovsk, abandoned airport 'Kirovsk', 33.58224°N, 67.57926°E, near buildings, 15.07.2020, *M. Kozhin* M-4412 (H, KPABG 46904, MW 1066862).

##### Pathways of introduction

Escape from confinement: Ornamental purpose other than horticulture.

This is a popular ornamental plant, which can survive for a long time after planting without further management.

##### Period of introduction

USSR, after the Second World War (1945-1991).

This is a popular garden plant of the Soviet times, which was known as capable to self-seed and persist in abandoned cultivation for a long time, but its subspontaneous occurrence has never been formally reported in floristic works in Murmansk Region.

##### Invasion status

The species was originally introduced in 1937 into the Polar-Alpine Botanical Garden and was known to self-seed around the places of original cultivation without spreading into other anthropogenic or native landscapes (Andreev and Zueva 1990).

Our record is a remnant of abandoned cultivation, similarly maintaining itself locally without expansion.

#### Ecology

Temperate forb forests.

#### Biology

Perennial polycarpic. Hemicryptophyte with short rhizome.

### Bromus commutatus

Schrad.

DAD2A9DE-062D-56DA-A423-E24901F7E66A

urn:lsid:ipni.org:names:393635-1

https://laplandflora.ru/#28335

https://www.gbif.org/occurrence/3400181308

Bromus commutatus Schrad., Fl. Germ. 1: 353 (1806).

#### Distribution

##### Native distribution

Mediterranean, western Asia, Caucasus, Iran.

##### Secondary distribution

Fully naturalised (archeophyte) in Atlantic and Temperate Europe. Casual in Northern Europe and Northern Asia; established in North and South America, Southern Africa, Australia.

##### Distribution in neighbouring territories

Rare casual in southern Finland (Hämet-Ahti 1998a), southern Karelia (Kravchenko 2007) and North-Western European Russia (Tzvelev 2000), most commonly found on railways or in places of discharge.

##### New record

Russia. Murmansk Region. Kandalaksha District. Kovda Village, collective farm 'Belomor', potato field in use of Demidov, solitary, 13.08.1953, *E. Shlyakova* #72 (KPABG 042581).

##### Pathways of introduction

Transport – Contaminant: Seed contaminant.

The species was found on fields, thus indicating its arrival with contaminated seed or planting material.

##### Period of introduction

USSR, after the Second World War (1945-1991).

This record is linked to the intensification of agriculture in the USSR after the war time. Its long-term survival in agricultural habitats is considered highly unlikely.

##### Invasion status

Historical casual occurrence. No new records, apparently extinct in the territory.

#### Ecology

Xerothermic meadows.

#### Biology

Annual. Therophyte with fibrous roots.

#### Notes

This record was misidentified by Shlyakova (1982) as *Bromusarvensis* L., but the collected specimen clearly differs from the latter species in the longer (up to 1 mm) pubescence on the leaf sheaths and the longer (5-8 mm) awns. Based on the compact racemes, the broadly angulate margin of lodicules and the larger (ca. 21 mm) spicules and (5-10 mm) lodicules, the specimen belongs to *B.commutatus* (Tzvelev 2000, Tzvelev and Probatova 2019).

One more taxon in this group, B.secalinussubsp.decipiens Bomble & H.Scholz or B.commutatussubsp.decipiens (Bomble & H.Scholz) H.Scholz, was recently separated in Central and Southern Europe (Bomble and Scholz 1999) and also reported from Sweden, Scandinavia (Valdés and Scholz 2009). This taxon is characterised by a less distinctly angulate margin of lodicules and does not correspond to our plant; so far, it has never been reported from Russia (Tzvelev and Probatova 2019).

The other specimens referred to *B.arvensis* by Shlyakova (1982) correctly belong to the species.

### Chaerophyllum hirsutum

L.

FD86F078-DC43-5CB2-BB1E-986498D09AC1

urn:lsid:ipni.org:names:840199-1

https://laplandflora.ru/#28298

https://www.gbif.org/occurrence/3400181309

https://www.gbif.org/occurrence/3400181310

Chaerophyllum hirsutum L., Sp. Pl. 1: 258 (1753).

#### Distribution

##### Native distribution

Europe (temperate, montane regions).

##### Secondary distribution

Northern Europe.

##### Distribution in neighbouring territories

Rare casual in southern Finland, apparently arrived with transport (Hämet-Ahti 1998b).

##### New record

Russia. Murmansk Region. Apatity Town. Northern part of Akademgorodok near the road along Kozlov Street, near the car depot, 33.39388°N, 67.57378°E, thickets of hogweed at the edge of the small-wooded willow, 23.06.2020, *M. Kozhin & E. Borovichev* M-4406 (H, KPABG 46898, KPABG 46899, MW 1066860, INEP).

##### Pathways of introduction

Escape from confinement: Ornamental purpose other than horticulture.

**Figure 3.**
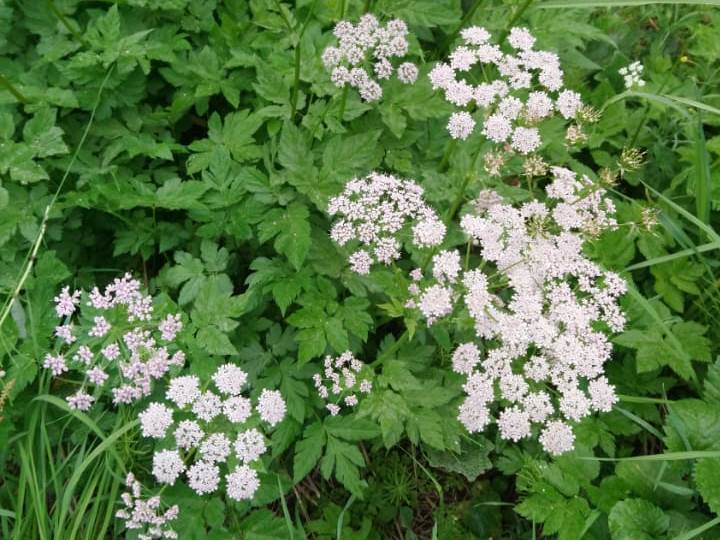
*Chaerophyllumhirsutum* in Apatity Town, Murmansk Region. 23.06.2020. Photo by E. Kopeina.

The species is a popular ornamental plant of recent times, cultivated in populated places as tall forb for flowers and foliage (Fig. 3).

##### Period of introduction

Russia (after 1991).

This introduction is firmly linked with the recent cultivation of this ornamental plant, which was not used in the USSR.

##### Invasion status

Persisting population in a man-made habitat (populated place).

#### Ecology

Riversides, moist forests.

#### Biology

Perennial polycarpic. Hemicryptophyte with caudex and short rhizome.

#### Taxon discussion

This species is represented by a cultivated variety with pink flowers, *Chaerophyllumhirsutum* 'Roseum'. Its garden origin is, therefore, beyond doubt.

### Galega orientalis

Lam.

AC30B776-F60B-5B3D-B13B-433F7CC9DA15

urn:lsid:ipni.org:names:495682-1

https://laplandflora.ru/#21395

https://laplandflora.ru/#28300

https://laplandflora.ru/#28301

https://www.gbif.org/occurrence/3400181311

https://www.gbif.org/occurrence/3400181305

https://www.gbif.org/occurrence/3400181301

Galega orientalis Lam., Encycl. 2(2): 596 (1788).

#### Distribution

##### Native distribution

Caucasus (Russia, Georgia, Armenia, Azerbaijan).

##### Secondary distribution

Information incomplete due to the recent time of invasion. Reported as commonly running wild and established in, for example, Finland (Niemivuo-Lahti 2012) and European Russia (Majorov et al. 2013).

##### Distribution in neighbouring territories

In Finland, this species commonly runs wild in the whole country, up to its northern part (LUOMUS 2021). It is considered a noxious weed and listed as a dangerous invasive species (Niemivuo-Lahti 2012). Sometimes it runs wild in central and southern Karelia (Kravchenko 2007) and North-Western European Russia (Tzvelev 2000).

##### New record

Russia. Murmansk Region. Apatity Town:

Polar Experimental Station of Institute of Plant Industry, 33.37094°N, 67.54942°E, field overgrown with dandelions, cereals and bedstraw, 23.06.2020, *M. Kozhin & E. Borovichev* M-4405 (H, KPABG 046897, MW 1066861).

Fields of the state farm 'Industry' at the entrance to Apatity Town, 33.32479°N, 67.57402°E, roadside between willow stands separating the fields, 500 m south-west of the road, 13.07.2020, *M. Kozhin* M-4408 (H, KPABG 046901, MW 1066864, INEP).

Fields of the state farm 'Industry' at the entrance to Apatity Town, 33.32641°N, 67.5765°E, road between fields, 13.07.2020, *M. Kozhin* M-4409 (H, KPABG 046902, MW 1066865, INEP).

##### Pathways of introduction

Escape from confinement: Agriculture. Escape from confinement: Research.

The species was cultivated as a forage plant and subsequently escaped from cultivation. In Murmansk Region, it was originally introduced into experimental cultivation (laboratory) in the Polar-Alpine Botanical Garden in 1939 (Andreev and Zueva 1990). By 1990, these plants were commonly found reproducing by seed around former cultivation places in the Botanical Garden.

**Figure 4.**
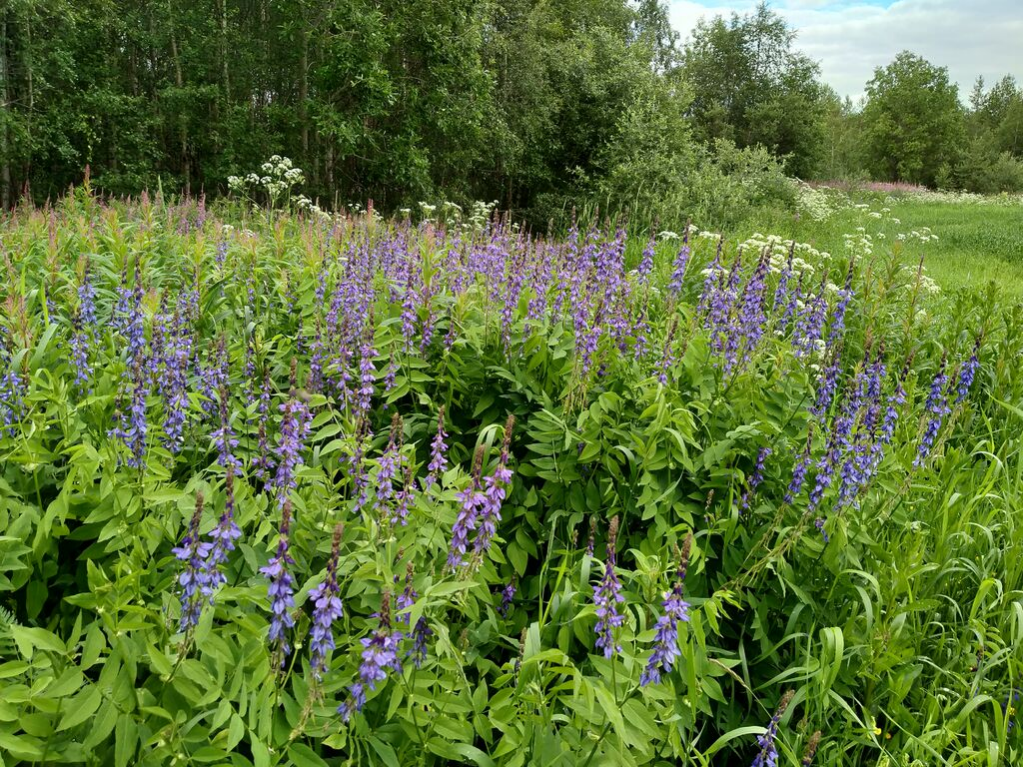
A dense stand of *Galegaorientalis* established in the vicinity of Apatity Town, Murmansk Region. 13.07.2020. Photo by M. Kozhin.

Since 1990, the experimental cultivation of *Galega* was carried out at the Polar Experimental Station of the Institute of Plant Industry and the new variety "Zapolarnyi" was bred. This variety was recommended for commercial cultivation in the northern agricultural regions of Russia (Mikhailova et al. 2011). The present record originated from the fields on which *Galega* was cultivated. Nowadays, the species occurs as extensive stands along abandoned fields and roadsides (Fig. 4).

##### Period of introduction

Russia (after 1991).

The species started to escape during the period of its commercial cultivation for forage, which became common in the latest 20 years.

##### Invasion status

Established alien, naturalised in anthropogenic habitats. Potentially invasive but not expanding into natural habitats.

#### Ecology

Tall forb of mountain meadows.

#### Biology

Perennial polycarpic. Hemicryptophyte with caudex and root sprouts.

### Geum aleppicum

Jacq.

06E1DDA3-5FC1-5318-9EF9-0098A1A6358A

urn:lsid:ipni.org:names:30094401-2

https://laplandflora.ru/#28329

https://www.gbif.org/occurrence/2028619743

Geum aleppicum Jacq., Collectanea 1: 88, t. 127 (1787).

#### Distribution

##### Native distribution

Eastern Europe (southern boreal and temperate), Northern Asia, North America.

##### Secondary distribution

Central and Northern Europe.

##### Distribution in neighbouring territories

In Finland, this species occurs as an established neophyte in the southern part of the country (Vuokko and Hämet-Ahti 1998). In Karelia, the species was found in scattered localities in the southern part and rarely in the northern part of the territory (Kravchenko 2007).

##### New record

Russia. Murmansk Region. Lovozero District. Revda Village (SW part), close to the museum buildings, 02.08.2011, *M. Piirainen* 6061 (H 827871).

##### Pathways of introduction

Transport – Stowaway: People and their luggage/equipment.

This zoochorous species is a ruderal plant commonly found along pedestrian paths. Revda is a large village with many people employed in mining, and with tourist attractions as, for example, a museum of local studies, near which the species has been found. We, therefore, assume that the plant was transported to the place of occurrence on people visiting the village.

##### Period of introduction

Russia (after 1991).

Considering the capability of this species to establish and spread further, we assume that its introduction was very recent.

##### Invasion status

According to the collector's notes, a sparse population of the species was observed. The species was considered as a locally established neophyte.

#### Ecology

Forest margins and meadows.

#### Biology

Perennial polycarpic. Hemicryptophyte with short rhizome.

### Leonurus quinquelobatus

Gilib.

827E4BE0-3E2C-596E-BD69-E446BE2190ED

urn:lsid:ipni.org:names:449227-1

https://laplandflora.ru/#16291

https://laplandflora.ru/#16292

https://laplandflora.ru/#16291

https://plant.depo.msu.ru/open/public/item/MW1058418

https://www.gbif.org/occurrence/2907937106

https://www.gbif.org/occurrence/2876127028

Leonurus quinquelobatus Gilib. in Usteri, Delect. Opusc. Bot. 2: 321 (1793).
Leonurus quinquelobatus *Leonurusvillosus*Leonuruscardiacasubsp.villosus
Leonurus quinquelobatus *Leonuruscardiaca*

#### Diagnosis

The species differs from *Leonuruscardiaca* L. s. str. by its calyces and stems with abundant long hairs throughout (vs. glabrous or sparsely pubescent along ribs) and lower cauline leaves deeply divided into narrow lobes (vs. dissected into broad lobes) (Gladkova and Menitsky 1978).

#### Distribution

##### Native distribution

Crimea, Caucasus, Iran.

##### Secondary distribution

Europe, Asia.

##### Distribution in neighbouring territories

Locally established neophyte in southern Finland (Kurtto 1998a) and southern Karelia (Kravchenko 2007).

##### New record

Russia. Murmansk Region. Terskii District. Kuzreka Village, near Botaminskaya fishing station, 66.598067°N, 34.834799°E, on a seashore meadow in a holiday village, 05.07.2018, *M. Kozhin* M-4036 (H, MW 1058418, KAND 10122).

##### Pathways of introduction

Escape from confinement: Ornamental purpose other than horticulture.

The species has been traditionally cultivated as folk medicine, although nowadays it practically fell into disuse and can seldom be found in cultivation.

##### Period of introduction

Russia (after 1991).

The record originated from a well-explored area, from which the species has not been known in the previous times. Its introduction is therefore considered recent.

##### Invasion status

Locally established neophyte, persisting but not spreading far from the original place of introduction.

#### Ecology

Mountain forests and shrublands.

#### Biology

Perennial polycarpic. Hemicryptophyte with short rhizome.

#### Notes

Kozhin (2014) reported the first occurrence of *Leonurusquinquelobatus* in Murmansk Region, which was based on a specimen collected from Umba Village. That plant was a misnamed specimen of *L.cardiaca* L. s.str.

### Lepidium densiflorum

Schrad.

3EF5A1D8-BC3B-544B-B49E-9DB57F0336D1

urn:lsid:ipni.org:names:286137-1

https://laplandflora.ru/#28328

https://www.gbif.org/occurrence/1948467413

Lepidium densiflorum Schrad., Index Seminum Horti Göttingen. 1832: 4 (1832).

#### Description

The species differs from the other species of *Lepidium* by the absence or near absence of petals, the absence of smell, larger fruits (ca. 3–3.5 mm long) in dense racemes (Tzvelev 2000), as well as stems and pedicels with very short capitate pubescence (D. German, pers. comm.).

#### Distribution

##### Native distribution

North America.

##### Secondary distribution

Europe, Asia, South America.

##### Distribution in neighbouring territories

Common and fully naturalised in southern Finland, rare casual in northern Finland (Suominen 1998). Rather rare but established in southern Karelia (Kravchenko 2007). Common and fully naturalised in North-Western European Russia (Tzvelev 2000), included in the list of most invasive plants in Russia (Vinogradova et al. 2018).

##### New record

Russia. Murmansk Region. Kandalaksha Town. SE side of the crossing of Ulitsa Gor'kogo and Ul. Pronina, surroundings of a gas station, 32.40583°N, 67.15888°E, sandy railway bank, 5.08.2011, *P. Uotila* 49222 (H 824080).

##### Pathways of introduction

Transport – Stowaway: Vehicles (car, train).

##### Period of introduction

Russia (after 1991).

This species was recorded from the place with intense transport activity, in current use. Its very recent introduction is therefore beyond doubt.

##### Invasion status

Only a few individuals were observed. The collector's notes suggested a casual occurrence.

#### Ecology

Open places, river sands, disturbed grasslands.

#### Biology

Annual (or overwintering biennial). Therophyte with taproot.

### Levisticum officinale

W.D.J.Koch

F3161853-65D1-56E7-818B-F7A74E36F7B5

urn:lsid:ipni.org:names:844187-1

https://laplandflora.ru/#19923

https://www.gbif.org/occurrence/3400181302

Levisticum officinale W.D.J.Koch, Nova Acta Phys.-Med. Acad. Caes. Leop.-Carol. Nat. Cur. 12(1): 101 (1824) - *Ligusticumlevisticum* L., Sp. Pl. 1: 250 (1753).

#### Distribution

##### Native distribution

Iran.

##### Secondary distribution

Europe, China, North America, South America.

##### Distribution in neighbouring territories

Established alien in southern Finland (Hämet-Ahti 1998b). Casual alien in Karelia, including the northern part (Kravchenko 2007).

##### New record

Russia. Murmansk Region. Kirovsk District. Highway Apatity - Kirovsk, 9th km, 33.55772°N, 67.58224°E, birch grass forest near the spring, 15.07.2020, *M. Kozhin* M-4410 (H, KPABG 046903, MW 1066866).

##### Pathways of introduction

Escape from confinement: Agriculture.

Frequently cultivated as a salad herb, a vegetable or a spice (lovage). This particular occurrence may be of secondary origin (arrived with relocated waste).

##### Period of introduction

Russia (after 1991).

It is uncertain how long-persisting this population is. The bad habit of placing garden and household waste along roadsides is relatively new in the Russian North, so we linked this record with the recent decades.

##### Invasion status

Locally established alien, introduced into natural habitats.

#### Ecology

Riversides.

#### Biology

Perennial polycarpic. Hemicryptophyte with caudex and short rhizome.

### Myrrhis odorata

(L.) Scop.

5C550C1F-116E-5E86-B01E-BDE934D64EB3

urn:lsid:ipni.org:names:845120-1

https://laplandflora.ru/#28299

https://www.gbif.org/occurrence/3400181304

Myrrhis odorata (L.) Scop., Fl. Carniol., ed. 2. 1: 207 (1771) - *Scandixodorata* L., Sp. Pl. 1: 257 (1753).

#### Distribution

##### Native distribution

Europe (temperate), Mediterranean.

##### Secondary distribution

Europe, North America.

##### Distribution in neighbouring territories

Naturalised in south-western Finland, casual in central Finland and southern Karelia (Hämet-Ahti 1998b, Kravchenko 2007).

##### New record

Russia. Murmansk Region. Apatity Town. Northern part of Akademgorodok near the road along Kozlov Street, near the car depot, 33.39388°N, 67.57378°E, thickets of hogweed at the side of a small-wooded willow, 23.06.2020, *M. Kozhin & E. Borovichev* M-4407 (H, KPABG 046900, MW 1066863).

##### Pathways of introduction

Escape from confinement: Ornamental purpose other than horticulture.

Cultivated as an ornamental plant for flowers and foliage.

##### Period of introduction

Russia (after 1991).

This is a place of recent cultivation of this ornamental plant, same as for *Chaerophyllumhirsutum*.

##### Invasion status

Persisting population in a man-made habitat (populated place).

#### Ecology

Mountain forb forest.

#### Biology

Perennial polycarpic. Hemicryptophyte with caudex.

#### Notes

**Figure 5.**
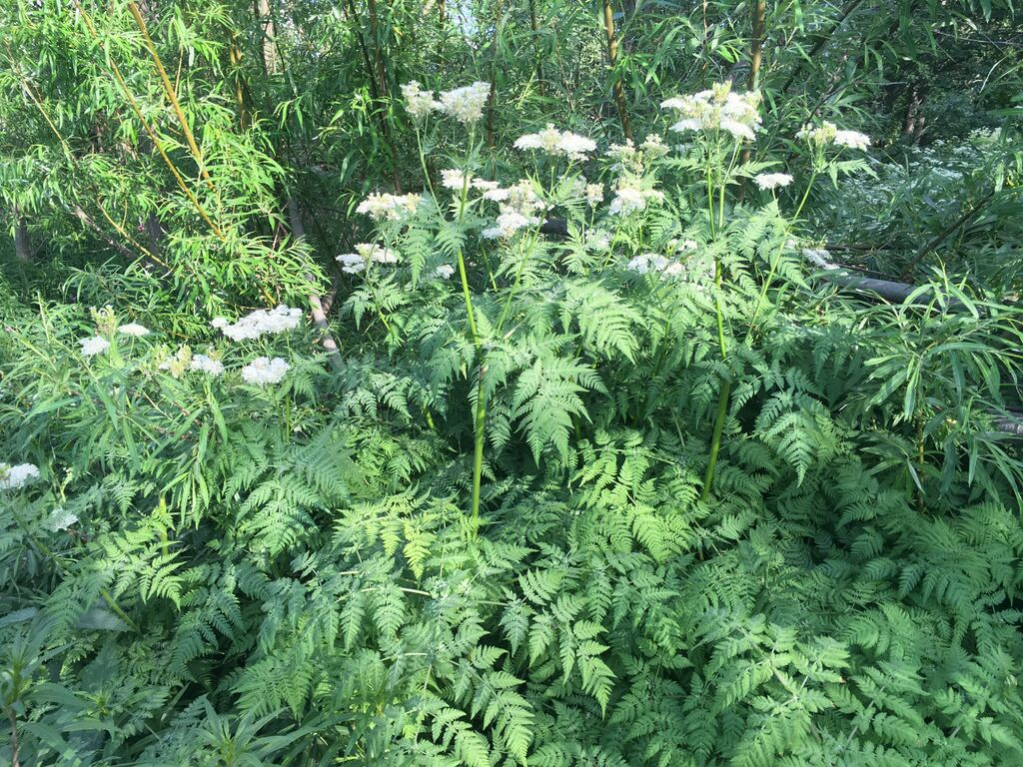
*Myrrhisodorata* in Apatity Town, Murmansk Region. 23.06.2020. Photo by M. Kozhin.

The species forms large stands (Fig. 5).

### Phleum phleoides

(L.) H.Karst.

EDC5506A-C031-529C-9AE5-EB5D48E5671A

urn:lsid:ipni.org:names:415866-1

https://laplandflora.ru/#28333

http://rr.herbariumle.ru/01128388

https://www.gbif.org/occurrence/3400241301

Phleum phleoides (L.) H.Karst., Deutsche Fl. 4: 374 (1881) - *Phalarisphleoides* L., Sp. Pl. 1: 55 (1753).
Phleum phleoides *Phleumboehmeri*

#### Distribution

##### Native distribution

Central and Southern Europe, Mediterranean, Eastern Europe (temperate), Northern Asia (temperate), Central Asia.

##### Secondary distribution

Northern Europe, northern part of Northern Asia (established), North America (casual).

##### Distribution in neighbouring territories

Archeophyte in south-western Finland, neophyte in south-eastern Finland (Hämet-Ahti 1998a). In Karelia, the species was recorded as a rare casual in ruderal or waste places since the Second World War up to the northern part of the territory (Kravchenko 2007). In the north-western part of Eastern Europe, the northern limit of its native distribution is situated in Pskov Region (Tzvelev and Probatova 2019).

##### New record

Russia. Murmansk Region. Khibiny Mts., vicinity of Khibinogorsk [Kirovsk] Town, wasteland on the north slope of Takhtarvumchorr Ridge, by the way from the bank of Malyi Vud'yavr Lake to Molybdenum Mine, 14.07.1934, *O. Polyanskaya* (LE 01128388).

##### Pathways of introduction

Transport – Contaminant: Contaminated bait.

The species was found in disturbed places along the road before the Second World War, thus indicating its possible import with hay.

##### Period of introduction

USSR, before the Second World War (1918-1941).

This occurrence is strictly casual and can be linked with the period of recording, when imported hay was still commonly used for local horse transportation.

##### Invasion status

This is a historical record of early casual occurrence. No new records, apparently extinct in the territory.

#### Ecology

This species is native to the steppe biome and occurs in grasslands.

#### Biology

Perennial polycarpic. Hemicryptophyte, laxly cespitose.

#### Notes

Although this specimen was deposited in a public collection and revised by all experts, it was not taken into account by the Flora of Murmansk Region (Kuzeneva 1953) or taxonomic reference books (e.g. Tzvelev and Probatova 2019).

The specimen was originally identified as *Phleumboehmeri* Wibel, which is a synonym of *P.phleoides* (Valdés and Scholz 2009).

### Prunus armeniaca

L.

AA9B5676-CFF4-515E-92AE-B9E527F9E6E2

urn:lsid:ipni.org:names:729463-1

https://laplandflora.ru/#28331

http://rr.herbariumle.ru/01127282

https://plant.depo.msu.ru/open/public/item/MW0384230

https://www.gbif.org/occurrence/3400711301

https://www.gbif.org/occurrence/1697533293

Prunus armeniaca L., Sp. Pl. 1: 474 (1753) – *Armeniacavulgaris* Lam., Encycl. 1(1): 2 (1783).

#### Distribution

##### Native distribution

Central Asia, China.

##### Secondary distribution

Europe (temperate), Mediterranean, Asia Minor, Caucasus, Iran, Australia.

##### Distribution in neighbouring territories

Previously, this species was frequently found as casual (young seedlings) along railway tracks in Karelia (Gusev 1971) and North-Western European Russia (Tzvelev 2000; Sennikov, pers. obs.).

##### New record

Russia. Murmansk Region.

Apatity Railway Station, northern outskirts, on a railroad track, 30.07.1970, *Yu. D. Gusev* (LE01127282);

Kandalaksha District. Poyakonda Railway Station, along the railway track, 24.08.1993, *A. Notov & D. Sokolov* (MW 0384230).

##### Pathways of introduction

Transport – Contaminant: Food contaminant.

The species has repeatedly arrived to the territory through waste from human consumption.

##### Period of introduction

USSR, after the Second World War (1945-1991); Russia (after 1991).

The species has been introduced many times and in many places through human waste. Its recording period corresponds to the times of the high availability and popularity of dried apricots.

##### Invasion status

Strictly casual, ephemerous. Juvenile individuals have been observed.

#### Ecology

Semi-arid mountain forest.

#### Biology

Tree. Phanerophyte.

#### Notes

The specimen collected by Gusev in 1970 had not been taken into account in a timely manner and was not included in Gusev (1971) or any subsequent publication.

### Rorippa sylvestris

(L.) Besser

19A296ED-96D2-56E6-98E1-509B67C255B5

urn:lsid:ipni.org:names:288692-1

https://laplandflora.ru/#28332

https://www.gbif.org/occurrence/3400181307

Rorippa sylvestris (L.) Besser, Enum. Pl.: 27 (1822) – *Sisymbriumsylvestre* L., Sp. Pl. 2: 657 (1753).

#### Distribution

##### Native distribution

Europe (boreal and temperate), Caucasus.

##### Secondary distribution

Europe (boreal), Mediterranean, Central Asia, Siberia, Russian Far East, North America.

##### Distribution in neighbouring territories

This species is naturalised in southern Finland (Suominen 1998) and North-Western European Russia (Tzvelev 2000). It is included in the list of harmful alien plants in Finland (Niemivuo-Lahti 2012). In Karelia, the species is known from a few scattered localities in populated places up to Kem Town in the north (Kravchenko 2007), where it probably persisted for a while.

Kotov (1979) reported this species from the Russian European Arctic, but his record was questioned by Dorofeev (2012). Wasowicz et al. (2019) omitted this publication as having no background literature or herbarium record.

##### New record

Russia. Murmansk Region. Kandalaksha District. Vicinity of Alakurtti Village, left bank of Tumcha River, along the shore of the stream, 25.07.1957, *O. Kuzeneva & A. Dryakhlova* 282 (KPABG 024968).

##### Pathways of introduction

Transport – Contaminant: Contaminated bait.

The species has arrived with forage transported to the place of occurrence by the German army during the Second World War.

##### Period of introduction

Second World War (1941-1945).

The period of introduction is established through the pathways.

##### Invasion status

Naturalised, locally established in native habitats. Historical occurrence.

The local population of this perennial colonist species persisted for at least 13 years by the time of its discovery. Its current state is unknown.

#### Ecology

Riversides, floodplains.

#### Biology

Perennial polycarpic. Hemicryptophyte with root sprouts.

#### Notes

In northern Finland (Kuusamo), *Rorippasylvestris* was recorded as locally established in a former German military camp, where it arrived to the territory during the Second World War with forage supply (hay) (Ahti and Hämet-Ahti 1971). We assume the same origin for the species occurrence in Murmansk Region, since the territory of Alakurtti Village was an important airfield and camping place for German military troops in the period of the German occupation in August 1941 - September 1944.

### Senecio vernalis

Waldst. & Kit.

F8BE1536-B8E9-52E8-8AC7-5BECE673DB76

urn:lsid:ipni.org:names:248179-1

https://laplandflora.ru/#28330

https://www.gbif.org/occurrence/1948436060

Senecio vernalis Waldst. & Kit., Descr. Icon. Pl. Hung. 1: 23, t. 24 (1800) - Senecioleucanthemifoliussubsp.vernalis (Waldst. & Kit.) Greuter, Willdenowia 33(2): 247 (2003).

#### Distribution

##### Native distribution

Europe (temperate), Mediterranean, Caucasus, Iran.

##### Secondary distribution

Europe (boreal, Atlantic), sometimes elsewhere in the world.

##### Distribution in neighbouring territories

Rare casual in Karelia, likely introduced during the Second World War (Kravchenko 2007). Casual alien in the whole of Finland (Kurtto 1998b).

##### New record

Russia. Murmansk Region. Pechenga District. 'Lapponia Petsamoensis, Jäniskoski, ent. leirialue', 22.08.1957, *C. E. Sonck* 6061 (H 761596).

##### Pathways of introduction

Transport – Contaminant: Contaminated bait.

The species has arrived with forage transported to the place of occurrence by the German army during the Second World War.

##### Period of introduction

Second World War (1941-1945).

The period of introduction is established through the pathways.

##### Invasion status

Locally established, persisting. Historical occurrence.

The local population of this annual species persisted for at least 13 years by the time of its discovery. Its current state is unknown, but presumably extinct.

#### Ecology

Open ground, disturbed ground.

#### Biology

Annual (or overwintering biennial). Therophyte with taproot.

#### Notes

The Jäniskoski-Niskakoski area was a territory in Inari Lapland, northern Finland, which was sold to the USSR in 1947 in order to establish a complex of hydropower electric plants for the needs of Soviet nickel mining projects in Petsamo District. The Finnish enterprise *Imatranvoima* was contracted to construct these electric plants and operated a few villages of Finnish construction workers in the territory, including Jäniskoski. Carl Erik Sonck, at that time a medical doctor and amateur botanist, served for the business and collected in this territory in the 1950s (Kravchenko 2020).

*Seneciovernalis* in Jäniskoski was collected in the place of a former German military camp, where it was introduced during the Second World War by German military troops in the period of the German occupation in August 1941 - September 1944. Other alien plant species of the same origin were collected in the same place, for example, *Erodiumcicutarium* (L.) L’Hér. (Mäkinen et al. 2019).

## Discussion

One half of the new records presented here are derived from historical collections, which are kept at major academic institutions in Helsinki (H), Apatity (KPABG), Moscow (MW) and Saint-Petersburg (LE). Although these collections have already been screened for overlooked occurrences (e.g. Kozhin et al. 2018), there is no surprise that further important specimens are being constantly unearthed as long as the comprehensive database of herbarium collections from Murmansk Region remains unfinished. During our data collection activity, aiming at the comprehensive databasing of plant specimens collected from Murmansk Region, we have recently found important records in the genus *Rosa* (specimens kept at H and S), which were overlooked during the data collection for the pan-European project *Atlas Florae Europaeae* (Khapugin et al. 2021). In that case, Russian specimens kept in the foreign collections were neglected because such collections have not been the focus of the foreign researchers, although these specimens can contribute significantly to floristic studies despite their small amount. Even more important omissions may occur when historical collections, kept remotely outside the country of origin, contain type specimens of local endemic taxa, which may go unnoticed and subsequently redescribed by the local botanists (e.g. Sennikov 2021, Lazkov and Sennikov 2021).

The historical occurrences of alien plants, newly reported here, appeared in the territory largely as contaminants (of seed or forage). Seed contamination had been declining already in the second half of the 20^th^ century (Suominen 1970, Shlyakova 1982), now being largely of historical importance. Forage contamination (arrival with imported hay) is a historical pathway, which was highly active when horses were commonly employed as transport power. This observation reflects a common trend of significant decline, both in frequency and diversity, of arable weeds (Fried et al. 2009), including archaeophytes (Preston et al. 2004).

Two historical records are of special importance, *Rorippasylvestris* and *Seneciovernalis*. Both species are currently known as widely distributed garden weeds or ruderal plants, whereas their occurrence in Murmansk Region is limited to the territory which was impacted by the Second World War. Such plants, called polemochores (Mannerkorpi 1944), typically arrived to Northern Europe and the north-western European part of Russia with hay imported by military troops (e.g. Ahti and Hämet-Ahti 1971, Kravchenko 2007, Sennikov 2012). In Murmansk Region, this type of historical introduction has been commonly neglected, but recent studies revealed the presence of such plants in the territory (e.g. Kostolomov 1984, Piirainen and Alm 2001, Kozhin et al. 2019, Kozhin et al. 2020b).

The remaining records reflect our field activities which aim at documenting the current process of plant invasions in the Russian North. All these recent occurrences originated by escape from confinement (ornamental purposes, horticulture, agriculture), reflecting the high diversity of modern cultivation practices in commerce and private gardens. Ornamental cultivation has been constantly increasing its role in global plant invasions (van Kleunen et al. 2018), whereas this factor and escape from confinement as a whole are major pathways in the history of European plant invasions (Pergl et al. 2020). Among the three species of ornamental plants reported here, one (*Aruncusdioicus*) is a highly popular and common ornamental since the late Soviet times, which should have technically been listed earlier. Our records of the other species (*Chaerophyllumhirsutum*, *Myrrhisodorata*) reflect the recent developments in Russian horticulture, which are apparently connected with the economic uprising and the corresponding development of trade and more sophisticated greening of urban areas and private gardens; the same phenomenon has been previously noted in Britain by Dehnen‐Schmutz et al. (2007). So far, such records are strictly casual and do not indicate any potential for invasion, merely contributing to the list of alien plants without any noticeable harm to the environment (e.g. Thomas and Palmer 2015).

Among the occurrences reported here, five alien species are considered casual and eight species are treated as locally established or persisting (for uncertain time). Only one species, *Galegaorientalis*, is considered fully established and capable of further spreading in the territory, although without invasive potential.

Due to unresolved uncertainties in the background material, we cannot provide the exact number of non-native vascular plants in Murmansk Region yet. So far, we estimate that it slightly exceeds 500 species, including archeophytes and the most recent neophytes.

## Supplementary Material

XML Treatment for Anthemis ruthenica

XML Treatment for Aruncus dioicus

XML Treatment for Bromus commutatus

XML Treatment for Chaerophyllum hirsutum

XML Treatment for Galega orientalis

XML Treatment for Geum aleppicum

XML Treatment for Leonurus quinquelobatus

XML Treatment for Lepidium densiflorum

XML Treatment for Levisticum officinale

XML Treatment for Myrrhis odorata

XML Treatment for Phleum phleoides

XML Treatment for Prunus armeniaca

XML Treatment for Rorippa sylvestris

XML Treatment for Senecio vernalis

9B13CF3D-4970-5F3B-BC64-4C7F84E59169 10.3897/BDJ.10.e78166.suppl1 Supplementary material 1 New records of non-native vascular plants in Murmansk Region, Russia Data type occurrences File: oo_619570.txt https://binary.pensoft.net/file/619570 Mikhail N. Kozhin, Alexander N. Sennikov
